# Recurrent Ameloblastoma: A Surgical Challenge

**DOI:** 10.1155/2018/8271205

**Published:** 2018-02-21

**Authors:** Chithra Aramanadka, Abhay Taranath Kamath, Adarsh Kudva

**Affiliations:** Department of Oral and Maxillofacial Surgery, Manipal College of Dental Sciences, Manipal, Karnataka, India

## Abstract

Ameloblastoma is locally aggressive benign odontogenic tumour with increased risk of recurrence rate. The choice of treatment depends on the histologic subtype. Radical therapy is the recommended modality for solid ameloblastomas. The possibilities of recurrence even after enbloc resection are still high. The author presents two case reports of recurrent ameloblastomas postradical resection. First case describes the recurrence of ameloblastoma in the bone graft which was used for reconstruction, and the second case depicts recurrence in the infratemporal fossa. Intraoperative radiography of the frozen section of the soft tissue margin plays an important role in the holistic management of these lesions.

## 1. Introduction

Ameloblastoma is the common locally aggressive benign epithelial odontogenic tumour of the oral cavity. It was first recognized by Cusack in 1827 and named in 1930 by Ivy and Churchill [1]. According to WHO classification in 2005, there are 5 subtypes of benign ameloblastoma documented, and they are (1) solid/multicystic type, (2) desmoplastic type, (3) unicystic type, and (4) extraosseous/peripheral type [2]. Histopathologically, the 6 subtypes are follicular, plexiform, acanthomatous, basal, unicystic, and desmoplastic ameloblastoma. It can be managed either by the conservative method or radical approach depending on the type, location, and size and age of the patient. A systematic review by Almaida et al. described that the 50% of recurrence is seen in follicular subtype and the recurrence rate is significantly low if a radical approach is used [3].

This paper describes two cases of recurrence of ameloblastoma in patients who underwent segmental resection of the jaw. These case reports can be added to the list of reported cases of recurrent ameloblastomas.

## 2. Case 1

**Figure 1:**
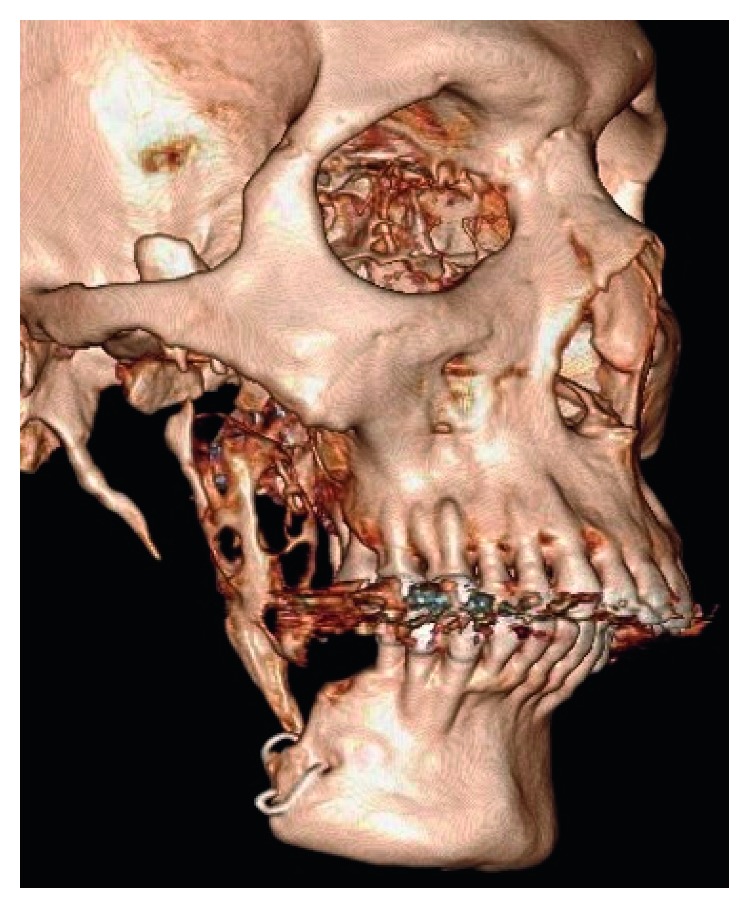
3-Dimensional reconstruction of CT imaging shows recurrent tumour of the grafted bone and remnant coronoid process.

**Figure 2:**
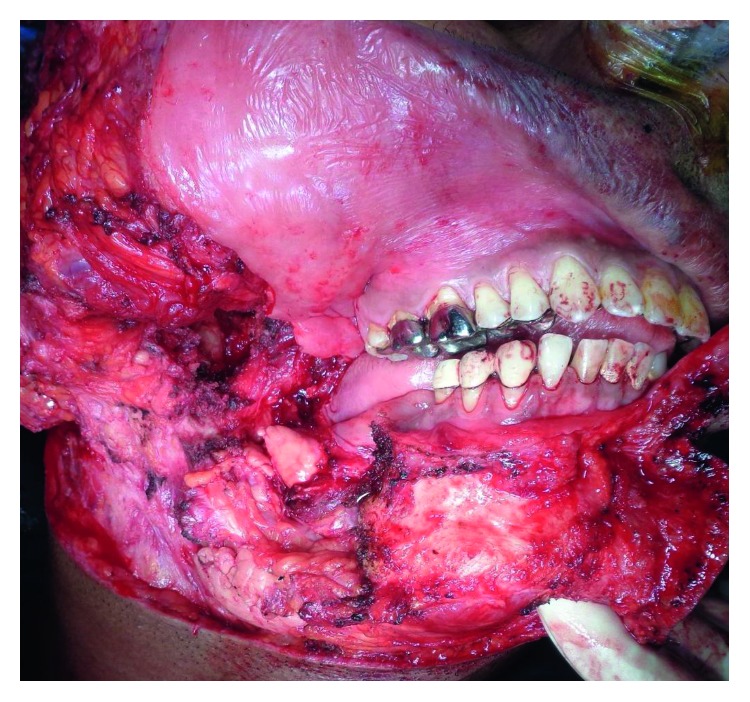
Surgical photograph shows the exposure of the lesion.

**Figure 3:**
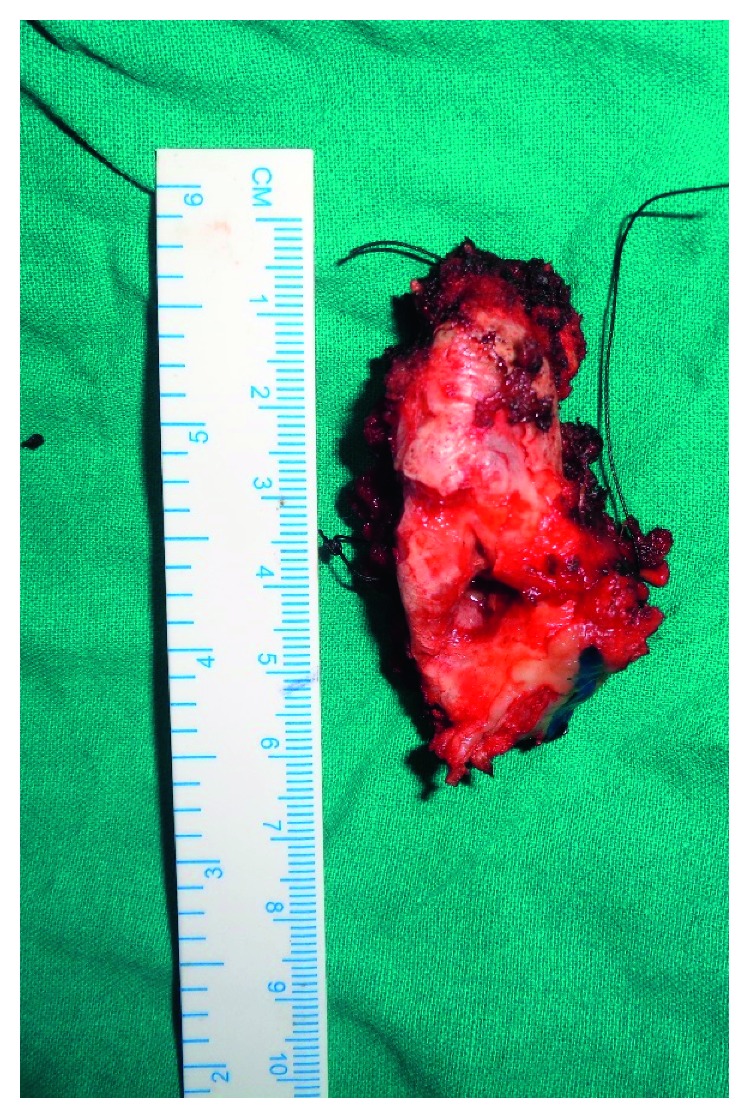
Surgical specimen.

A 46-year-old male patient referred by a private practitioner complained of swelling in the previously operated area of the right lower jaw since one month. He had a history of surgery in the same region. While going through the records of the patient, he had undergone segmental resection and reconstruction of the defect with rib graft 15 years ago. Histopathology reports of the previous pathology were not available in the records. Panoramic radiograph and CT scan revealed multilocular radiolucent lesion in the previously operated site (Figure 1). The clinical diagnosis at present was recurrent multicystic ameloblastoma involving the bone graft. The excision of the lesion with 1 cm uninvolved soft tissue margin was performed through the previous scar (Figures 2 and 3). The histopathology report of the specimen suggested follicular ameloblastoma with acanthomatous changes with tumour-free margins. A 1-year-follow-up showed no recurrence. He is planned for alloplastic reconstruction of the right hemimandible, considering the benign nature of the lesion.

## 3. Case 2

A 45-year-old male patient visited the Department of Oral and Maxillofacial Surgery with swelling over the right temple area of the face. He reported an asymptomatic swelling since 1 month which progressively increased in size. It was soft dumbbell shaped swelling in the right temporal region with no signs of infection.

Back in 2012, he had been referred to the same unit by general practitioner for gross swelling in the right jaw. Panoramic radiograph and CT scan showed multilocular radiolucency. The patient was subjected for biopsy and reported as ameloblastoma of the right mandible. He underwent right hemimandibulectomy. On table, the resected specimen was subjected for intraoperative radiography to understand the clearance of 1.5 cm safe radiologic margin. The histological report also revealed ameloblastoma with atypical features showing hypercellularity. Hence, he was kept on regular follow-up.

**Figure 4:**
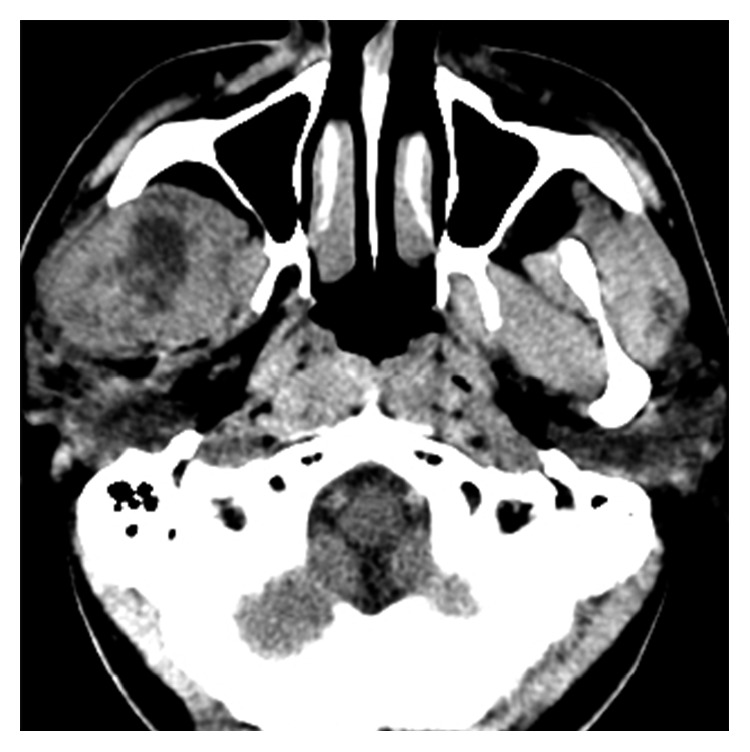
Preoperative CT scan shows the recurrent ameloblastoma in the infratemporal fossa.

**Figure 5:**
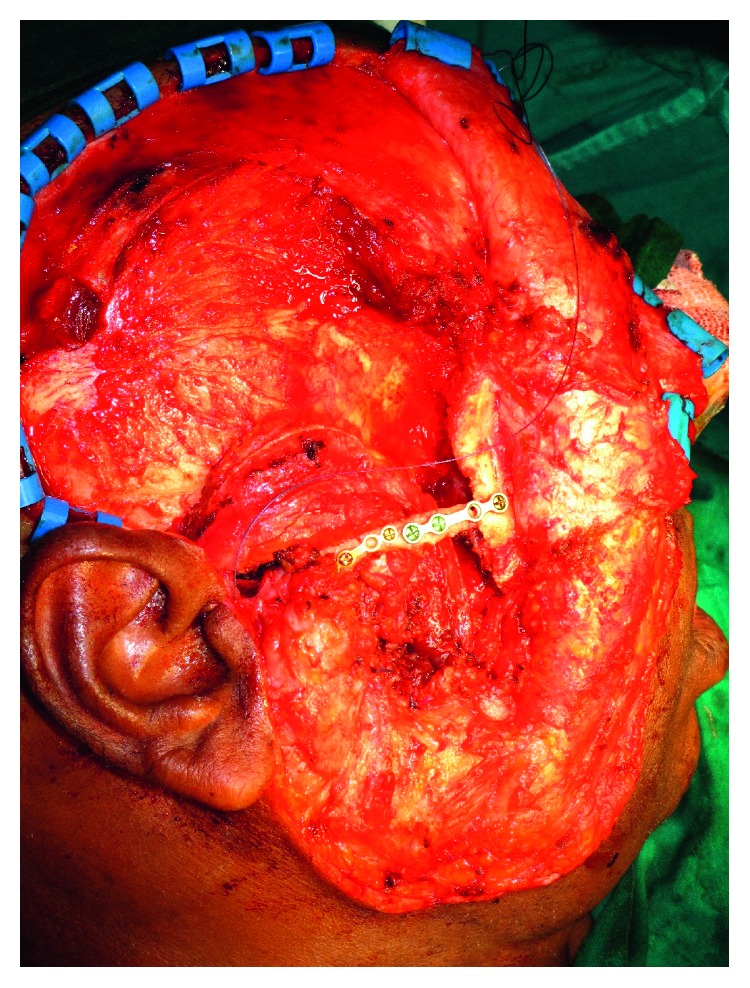
Intraoperative photograph shows the transzygomatic approach and the defect restored with temporalis muscle.

**Figure 6:**
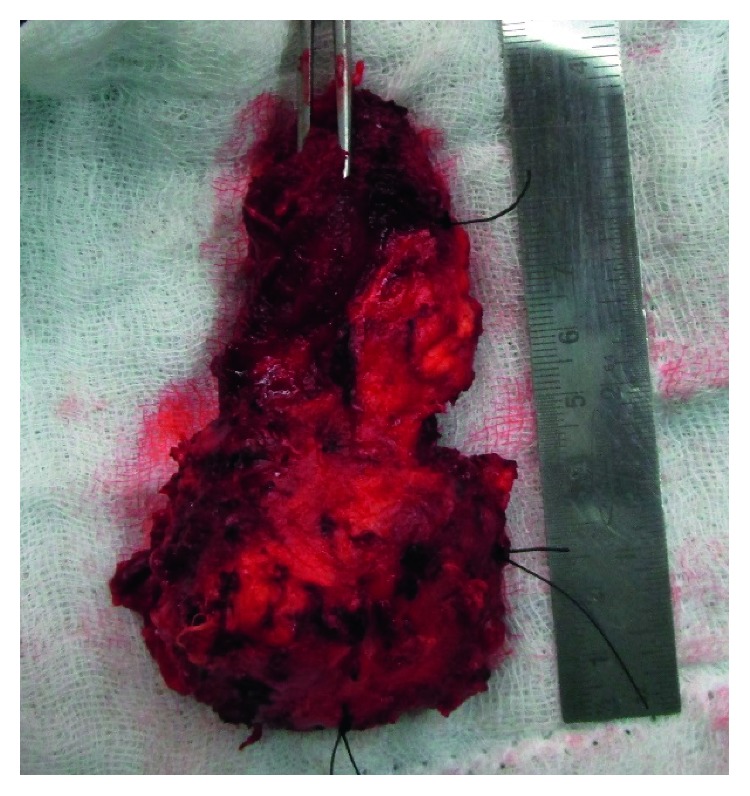
Excised specimen.

In 2016, he reported with a swelling on the right temple region. MR imaging studies showed heterogeneously enhancing altered signal intensity lesion (measures 4.4 cm × 3.7 cm × 4.6 cm) with tiny cystic areas noted in the right infratemporal fossa (Figure 4). The patient underwent tumour excision with a layer of overlying soft tissue through transzygomatic approach (Figures 5 and 6). The defect was packed with temporalis muscle flap. The specimen was sent for pathologic evaluation. Frozen sections confirmed the diagnosis of follicular ameloblastoma with a tumour-free margin. His hospital stay was uneventful, and he is currently on regular follow-up.

## 4. Discussion

Ameloblastoma is a locally aggressive, anatomically benign tumour of the oral cavity which rarely undergoes malignant transformation. It is the second most common odontogenic tumour, the first being odontoma. Ameloblastoma is a common tumour in developed countries (70%) compared to developing countries, probability of unreported case in the latter [4].

WHO in 2005 classified ameloblastoma into four subtypes: multicystic/solid, unicystic, desmoplastic, and extraosseous type. Solid/multicystic variant is the most common type, and it is highly aggressive and has a 90% recurrence after conservative management such as curettage and enucleation [5]. The high rate of recurrence is observed in mandibular ameloblastomas than maxillary ameloblastomas [6] and in follicular type than in plexiform or any other type [3].

Pathogenesis of the ameloblastoma is unclear. The genetic theory explains the involvement of the BRAF protein in the mitogen-activated protein kinase pathway (MAPK) that has been commonly found to be mutated, rendering the pathway constitutively active [7]. Ahlem et al. observed that the cell proliferation activity evaluated by Ki67 and CD10 was significantly higher in recurrent tumours [6].

More commonly, it affects the mandibular posterior region. The infiltration of the tumour cells occurs more predominantly in the cancellous portion of the cortical bone. Hence, CT scan is most promising in identifying the cortical destruction and soft tissue involvement [8].

The management involves either conservative or radical approach. The conservative method involves enucleation with adjunctive procedure either chemical cauterization or peripheral ostectomy of 1–1.5 cm normal margin. Conservative approach can be utilized for unicystic type. According to Pogrel and Montes, a unicystic variant can be best managed by enucleation with an application of Carnoy's solution or cryotherapy [9].

Radical approach is indicated for large ameloblastoma involving the inferior alveolar canal or below or for more aggressive variants like intramural ameloblastoma or multicystic type [10, 11]. It involves segmental or marginal resection with 1.5–2 cm normal bony margin beyond the radiologic margin. Use of intraoperative radiographs has been advocated for confirmation of bone margins.

The resection with 2-3 cm clear bone margin is indicated in cases of ameloblastic carcinoma [12].

Possible factors for recurrence after radical resection may be the histologic type and location of the tumour and solid type particularly follicular variety is the most aggressive type. The mandible posterior region is the common site of occurrence, and as it invades the cancellous portion beyond radiologic margin, over a time it can cause the cortical perforation. Invasion of the periosteum can lead to spread of the tumour cells to the soft tissue. Inadequate resection of the hard and soft tissues beyond a tumour would cause recurrence [13].

Several reports are available in literature related to the recurrence of ameloblastoma as shown in Table 1.

Recurrences are described in the study of radical treatment of ameloblastoma by Sehdev et al. [14], Shatkin and Hoffmeister [16], Mehlisch et al. [17], Muller and Slootweg [18], Olaitan et al. [19], Ueno et al. [20], Eckhardt et al. [10], and Nakamura et al. [21].

It is the known fact that the rate of recurrence with the conservative management is high (around 60%) compared to radical treatment (13%). The pattern of recurrences postresection has to be evaluated in a larger extent. Reports have been suggested that there are higher possibilities of retained soft tissue tumour islands during the surgical procedure in the complex regions like infratemporal fossa [34, 35].

In the first case report, the grafted tissue underwent tumorigenesis. This could be due to the remaining cells at the osteotomy site. It would be wise to wait for the histological report with the free margin before reconstruction in any case of the solid type to avoid donor site morbidities. The complication of primary reconstruction should be explained to the patient before surgery.

Lesions of infratemporal fossa remain asymptomatic, and the chief complaint of the patient is usually facial swelling or deformity.

Infratemporal fossa is pyramidal in shape that consists of complex structures, located on the lateral aspect of the cranial base, deep to the zygomatic arch, masseter, and mandibular ramus. The base formed by the medial aspect of the ramus and floor of the skull forms the upper surface of the pyramid. The anteromedial aspect corresponds to the posterior aspect of the maxilla and the posteroinferior aspect to the pterygomaxillary fascia [36]. It is anatomically confined, making it relatively inaccessible and allowing undetected neoplastic growth. A close approximation to vital structures such as the calvaria, nasopharynx, and the maxillary artery add to its anatomic complexity. Diagnosis and treatment planning should follow CT scan and MRI to determine the extent of the lesion in such areas. Conventional radiography may not provide sufficient information.

Numerous surgical approaches have been employed to access the infratemporal region, some of them being the transoral, transanal, transpalatine, transzygomatic, transcervical, and extended maxillectomy approach [37]. We found that transzygomatic approach was appropriate to approach the lesion and to harvest the temporalis flap into the defect. The choice of surgical approach to each type of neoplasm depends on the clinical presentation and histologic subtype as well as its extent and location.

A possible explanation for recurrence in the second case would be retained periosteum over the coronoid process. Composite resection would eliminate the retention of a tumour infiltrated tissues.

Carlson and Marx described the technique of excising the next uninvolved anatomical structure to prevent recurrence of a tumour. We do agree with his opinion of scientific approach towards curative management with histopathologic free soft and hard tissue margin [11]. Similar cases of recurrence of reconstructed bone have been reported in the literature following 30 years of surgery [25–32, 38–40].

Laborde et al. and Becelli et al. reported no recurrence in his study of 7 patients' postsegmental resection [41, 8]. Similar reports are documented by Vayvada et al., Chaine et al., and Basat et al. on postresection and free flap reconstruction [13, 15, 34, 42].

Vaishampayan et al. suggested the possible cause for recurrence in the infratemporal region after segmental resection would be due to the retraction of pathologically weakened coronoid process fragment during temporal dissection [43].

Peacock et al. concluded that there no additional benefit in confirming the margin by performing frozen section in addition to intraoperative specimen radiograph in his study of 35 patients. However, this study did not include the soft tissue margin [44].

Though several reports suggest the radio resistant nature of ameloblastoma, I^125^ brachytherapy is tried to irradiate the recurrent lesion by delivering high prescribed doses of radiation (110 Gy) with satisfactory outcome [45].

We propose few guidelines in the management when dealing with ameloblastoma eroding the cortical borders:

1. MRI study should be done in large ameloblastomas to evaluate the infiltration of the tumour into the adjacent soft tissue planes.
2. Intraoperative radiography should be done to rule out positive hard tissue margin.
3. Compartmental resection of the tumour should be performed to involve all positive margins and on table frozen section of the soft tissue margins [46].
4. Reconstruction should be performed as staged surgery in giant ameloblastomas after the complete histopathology report is available.
5. Long-term follow-up with MRI and CT imaging should be conducted after the primary surgery to evaluate any form of recurrence.

## 5. Conclusion

Our experience with two case reports suggests high local aggressiveness of the solid type of ameloblastoma. Tumour-free soft tissue margin in three dimensions should be considered when treating large lesions with an erosion of cortical outline. Clinical and histological study in larger extent will provide added information in the management.

## Figures and Tables

**Table 1:** 

Case Report	
Grafft et al. [22, 23]	Mandibular molar region; iliac graft
Carvalho et al. [24]	Iliac graft
Dolan et al. [25]	Rib graft
Marinelli et al. [26]	Iliac graft
Stea [27]	Iliac graft
Zacharides [28]	1 case of iliac graft; 2 cases of rib graft
Vasan [29]	Iliac graft
Bianchi et al. [30]	Iliac graft
Martins and Favaro [31]	Iliac graft
Su et al. [32]	Iliac graft
Choi et al. [33]	Iliac graft
Jian et al. [23]	1 case of iliac graft; 1 case of rib graft
Basat et al. [13]	1 case of free fibula flap
